# An audit of the use of CT pituitary scans to exclude a pituitary macroadenoma

**DOI:** 10.1186/s40842-023-00157-2

**Published:** 2023-12-15

**Authors:** Lisa Douglas, James Caldwell, Mark Bolland

**Affiliations:** 1 Endocrinology Department Greenlane Clinical Centre, Auckland City Hospital, Auckland, New Zealand; 2 Neuroradiology Department, Auckland City Hospital, Auckland, New Zealand

**Keywords:** Computerised tomography (CT), Pituitary macroadenoma, Magnetic resonance (MR)

## Abstract

**Background:**

Pituitary imaging is often required to exclude an adenoma suspected clinically or biochemically. Although magnetic resonance (MR) is the gold standard, computerised tomography (CT) is faster, cheaper and induces less claustrophobia. Our audit at Auckland City Hospital, New Zealand, investigated whether the use of CT of the pituitary as the first line imaging to assess for a pituitary macroadenoma reduces the need for MR.

**Methods:**

We investigated the usefulness of CT pituitary imaging in the exclusion of pituitary macroadenoma between 2012 and 2020. A re-audit was then undertaken for a period of one year between March 2021 and March 2022 to assess outcomes once a departmental policy change was implemented. At Auckland City Hospital, 32 patients across eight years were eligible for this analysis, of which 31 had data available. In our re-audit, 29 patients were eligible for this analysis. We collected data on patient demographics, relevant hormone levels, indication for imaging and imaging results and subsequent management.

**Results:**

After CT pituitary imaging, 28/31 (90%) of patients did not require further imaging because the clinical question had been addressed. One year after routine initial CT pituitary imaging was implemented by the Auckland City Hospital Endocrinology Department, 29 CT pituitary scans were performed to exclude a pituitary macroadenoma. Of these patients one required further imaging due to the finding of an expanded pituitary sella but not a pituitary macroadenoma.

**Conclusion:**

CT pituitary imaging to exclude a pituitary macroadenoma is a useful test that may reduce the need for MR pituitary scans.

**Trial registration:**

Not applicable. This was an audit as defined by the New Zealand National Ethics Advisory Committee guidelines. Please see ‘Declarations’ section.

## Background

Magnetic Resonance (MR) is the gold standard imaging modality for assessment of the pituitary gland because of superior definition compared to other options such as Computed Tomography (CT) [1]. This is particularly important for the recognition of small lesions such as microadenomas (by definition, tumours < 1 cm), for which the spatial and contrast resolution of CT is insufficient to allow reliable diagnosis [2]. Along with incidental diagnosis on brain imaging, pituitary adenomas are most commonly identified in specific pituitary imaging requested in the clinical setting of hormone excess (for example Cushings disease or acromegaly) or compressive symptoms (such as bitemporal hemianopia) [3]. In autopsy and radiology surveys, pituitary adenomas have a prevalence of about 17%, [3] but clinically relevant adenomas have a prevalence of 0.09% [4]. The incidence of pituitary adenomas is approximately 5 cases per 100,000 per year [4]. In prevalence studies, the overwhelming majority of tumours were < 1 cm, with about 1 in 100 pituitary adenomas meeting the definition for macroadenomas (tumours > 1 cm) [3]. However, the proportion of macroadenomas in clinically relevant pituitary adenomas is higher: an Argentinian retrospective cohort study including 2.8 million patients concluded 3/5 of adenomas detected were microadenomas [5]. Prolactinomas are the most common of the adenoma subtypes, followed by non-functional and then somatotrophinomas [4]. Most pituitary microadenomas remain stable, do not grow over time and do not cause compressive symptoms. By contrast, macroadenomas are more likely to grow and cause compressive symptoms and visual compromise.

In a number of clinical situations, identification of pituitary microadenomas is not necessarily required, because the key clinical question is to rule out a macroadenoma. Some examples include women with a persistent mildly elevated prolactin; in patients taking long-term dopamine antagonists with the expected elevated prolactin where the question of a possible underlying pituitary adenoma as the cause has been raised and the dopamine antagonist cannot be withdrawn (for example antipsychotic medication); or in men with low testosterone where a pituitary adenoma causing secondary hypogonadism as the cause has been suggested. In all of these situations, identification of a macroadenoma would change management whereas identification of a microadenoma would not. For the first scenario of persistent hyperprolactinaemia a trial of Cabergoline can be undertaken with or without a microadenoma; for the second and third scenarios, after excluding hormone excess, a microadenoma is likely an incidental lesion requiring no action or follow-up.

As a CT scan with dedicated pituitary reformats can readily rule out the presence of a macroadenoma, it presents an alternative option to MR in these scenarios and has some specific advantages. It does not require administration of contrast to exclude a macroadenoma, and induces less claustrophobia than a MR scan. When compared to MR pituitary, CT scans take less time (5 min vs. 35 min) and are associated with less cost. The current chargeable costs at Auckland City Hospital, to a non-resident, are $845 for a CT pituitary scan with contrast versus $1478 for an MR scan (Auckland City Hospital, Radiology Department Business Support Accountants Department, personal communication). Using CT rather than MR potentially also avoids patient anxiety associated with the diagnosis of an incidental microadenoma.

Currently, at Auckland City Hospital the demand for MR scanning outstrips the available resource, leading to longer waitlists. This situation has been exacerbated by the cessation of scanning during the coronavirus lockdowns. Currently the Radiology Department aims to perform MR pituitary scans within 6 weeks as a key performance indicator (KPI). CT pituitary scans usually occur within a few weeks. Attempting to achieve this MR KPI requires substantial outsourcing of MR to private providers, and our clinical experience has been of patients waiting much longer than 6 weeks for scans. Consequently, Endocrinologists at Auckland City Hospital have been requesting CT pituitary imaging more frequently but this practice has not been formally assessed.

Our objective was to establish if we could use CT pituitary imaging to exclude pituitary macroadenomas in our patients.

## Methods

Data for all patients in our initial audit who had CT pituitary scans ordered through Auckland City Hospital from January 2012 until October 2020 were obtained from the Picture Archiving Communication System (PACS). Some patients had their scan listed as a CT head and some had their scan listed as a CT pituitary but all had pituitary reformats, with or without contrast. We excluded patients with established pituitary pathology who had a CT as a follow-up to previous CT or MR imaging and scans not requested by an Endocrinologist. As a result of this audit, from March 2021 onwards, the Endocrinology Department at Auckland City Hospital requested CT pituitary scans as first line imaging to exclude pituitary macroadenomas. Our re-audit had the same inclusion and exclusion criteria and was conducted during the timeframe March 2021 until March 2022.

We extracted the following details from the online clinical records and imaging request form: patient demographics, symptoms and medication, scan details including indication and requestor department, relevant hormones prior to scan of prolactin (monomeric where measured) and testosterone, result of CT, whether MR was required, and subsequent treatment recommended.

This is an audit as defined by the New Zealand National Ethics Advisory Committee guidelines and therefore it did not require ethical approval [6]. Methods were carried out in accordance with these guidelines and informed consent was not required from subjects and therefore not obtained.

## Results

### Initial audit

The initial audit identified 31 eligible CT pituitary scans. The baseline characteristics of these patients are summarised in Table 1. During 2020, 13/31 (42%) were performed and overall 20/31 (65%) were female. The median age was 32 years (40 years for men and 29 years for women). The indications for imaging were hyperprolactinaemia (17/31, 55%), secondary hypogonadism without hyperprolactinaemia (11/31, 36%, 8 male, 3 female), visual disturbance (1/31, 3%), and investigation of acromegaly (2/31, 7%). Two patients had relative contraindications to MR imaging (they had pacemakers) – one with visual disturbance and one with suspected acromegaly.

**Table 1:** Baseline characteristics

	Original Audit	Re-audit
**Number of Patients**	31	29
**Gender**	Female 65% Male 35%	Female 69% Male 31%
**Median age**	32 years (29 years Female, 40 years Male)	33.5 years (34 years Female, 34 years Male)
**Indication for imaging**		
Hyperprolactinaemia	55%	69%
Secondary hypogonadism without hyperprolactinaemia	36%	21%
Visual disturbance	3%	0%
Other	6%	10%

#### Final diagnoses: Table 2

**Table 2:** Final diagnoses of audit patients

Diagnosis	Original audit: Number of patients	Re-audit: Number of patients
Hyperprolactinaemia		
Possible microprolactinoma and treatment commenced	6	5
Possible microprolactinoma or idiopathic and not requiring treatment	5	13
Pituitary macroadenoma	3	0
Medication related/related to another medical problem	2	2
No specific diagnosis made	1	0
**Secondary Hypogonadism, without hyperprolactinaemia**		
Hypogonadism secondary to drugs, obesity or excessive exercise	6	2
Related to another medical problem (PCOS, previous orchidectomy)	2	0
Idiopathic hypogonadism	3	4
**Other**		
Acromegaly	1	1
Post-concussion headache	1	0
No endocrine cause	1	1
Medication related	0	1

##### Hyperprolactinaemia

Of the seventeen patients (16 female, 1 male) for whom the indication for CT was an elevated prolactin, the median prolactin was 1257 mIU/L (range 520–2695 mIU/L). One patient had a macroadenoma diagnosed but no further MR imaging because of obesity, and two had abnormalities seen on CT for which MR imaging was recommended to further categorise: one was a macroadenoma and one a microadenoma. Thus, in 14/17 (82%) patients with hyperprolactinaemia, CT pituitary was able to provide sufficient information to obviate the need for MR imaging.

##### Secondary hypogonadism

Eleven patients had a CT pituitary for hypogonadism and did not have a raised prolactin. In the eight men, the median testosterone level was 6.9 nmol/L (range 4.7–8.6 nmol/L, lower limit of reference range 8.7 nmol/L for men aged 20–50 years). All 11 CT pituitary scans were normal; thus MR imaging was obviated in 100% of these patients.

##### Other indications

One patient had a CT pituitary scan because of visual field loss (bitemporal hemianopia). This patient had a macroadenoma identified and later had MR imaging although this was delayed for other medical reasons. Two patients had a CT pituitary for investigation of suspected acromegaly. One patient had a microadenoma confirmed but had no MR imaging because of a pacemaker. The other patient had the diagnosis of acromegaly raised during a hospital admission and had the CT performed prior to Endocrinology review because of other neurological symptoms. Hormonal testing did not show growth hormone excess.

Overall, 5/31 patients had an abnormal CT pituitary and the remaining 26/31 (84%) patients with normal CT scans did not require further imaging with MR to establish a management plan. Of the five patients with abnormal scans, three underwent MR, and two did not because of relative contraindications to MR.

### Follow-up re-audit after 1 year

Based on these results, the Auckland City Hospital Endocrinology Department introduced a policy of requesting CT pituitary scans as the first line investigation where the key clinical concern was to exclude a pituitary macroadenoma and we re-audited the results after one year.

In the follow-up audit, baseline characteristics are again summarised in Table 1. There were 29 eligible CT pituitary procedures identified. 20/29 (69%) were female. The median age was 34 years for both women and men. The indications for imaging were hyperprolactinaemia (20/29, 69%), secondary hypogonadism without hyperprolactinaemia (6/29, 21%, 5 male, 1 female), investigation of acromegaly (1/29, 3%), suspected central hypothyroidism (1/29, 3%) and suspected diabetes insipidus (1/29, 3%).

#### Final diagnoses: Table 2

##### Hyperprolactinaemia

Of the 20 patients (15 female, 5 male) for whom the indication for CT was an elevated prolactin, the median prolactin was 820 mIU/L (range 330–1713 mIU/L). In 100% of patients with hyperprolactinaemia, CT pituitary imaging was able to provide sufficient information to obviate the need for MR imaging.

##### Secondary hypogonadism

A CT pituitary scan was arranged for hypogonadism for six patients who did not have a raised prolactin. In the five men, the median testosterone level was 6.3 nmol/L (range 4.7–6.5 nmol/L). All six CT pituitary scans were normal and an MR scan was not required.

##### Other indications

One patient had a CT pituitary for suspected central hypothyroidism (normal), one for possible diabetes insipidus (normal), and one for suspected acromegaly which showed an expanded pituitary sella but no pituitary mass. This patient could not tolerate an MR scan and ongoing management and treatment is currently being considered.

In the re-audit overall, CT imaging excluded a macroadenoma in all patients, and in only one patient was further imaging with MR considered to establish a management plan.

## Discussion

This audit shows that in the specific setting where the key clinical question is the exclusion of a pituitary macroadenoma on radiological imaging, CT pituitary scans perform well as the initial imaging modality. In the initial audit, 84% of patients had a normal CT in this situation and did not require further imaging for their clinical management, and in the follow-up audit, all CT pituitary excluded a macroadenoma. Of the five patients in the initial audit with abnormal scans (two macroadenoma, one microadenoma, two abnormalities better characterised on MR), two did not have a follow-up MR because of relative contraindications. Only one patient in the re-audit required a follow-up MR but they were unable to tolerate it. The results suggest that it is reasonable to request a CT pituitary when the indication is to rule out a macroadenoma, obviating the need for a MR.

The main advantage of a CT pituitary for patients is in timing. If MR imaging was freely available with no time advantage for CT, then it would be the test of choice. However, when there are marked differences in wait times, the long delay before the diagnostic test can be obtained needs to be balanced against the extra information gained from the test. The current coronavirus pandemic has also put more pressure on limited hospital resources and requires alternative approaches that safely adjust workload to account for this. In addition, CT scans are faster, cheaper, and are better tolerated.

Referrals for hyperprolactinaemia are common. The key clinical concern is to exclude pituitary stalk compression from a macroadenoma or other mass lesion as the cause of the elevated prolactin. CT imaging allows this to be done, thereby permitting patients to be stratified into groups who either require further imaging and/or surgical assessment or those whose treatment (usually Cabergoline) can be initiated immediately.

The main disadvantages for the patient is that CT involves exposure to ionising radiation whereas MR does not, and that MR has greater spatial and contrast resolution than CT meaning that small lesions may not be detected by CT. Specific data on ionising radiation from a CT pituitary is difficult to find, however an approximate figure of 2 mSv (millisievert) radiation dose (range of 1–15 mSv) can be expected from a CT head scan [7]. This is a smaller dose than other common CT imaging procedures such as non-contrast CT chest (6 mSv) or abdomen (8 mSv), but more than a plain extremity radiograph (0.001 mSv) or chest film (0.1 mSv) [8]. Putting this in perspective, 2 mSv is about the same as the annual background radiation dose in New Zealand [9]. The ability of MR scans to detect anatomy in more detail than CT is well known. While MR pituitary scans provide better definition and will find more abnormalities, the clinical relevance of some of these can be debated. Some additional findings on MR scan are unlikely to change patient management further to that of the clinician’s judgement. For example, patients with a mildly raised prolactin with associated clinical symptoms can be treated regardless of whether there is a microprolactinoma (with MR imaging done prior to Cabergoline withdrawal if appropriate). An incidental finding of a microprolactinoma can lead to patient anxiety, and repeated MR imaging and hormone testing.

While MR pituitary scans are considered gold standard there is little research directly comparing MR and CT pituitary scans with each other specifically on the ability to detect a pituitary macroadenoma. Moreover, most of the literature that does exist comparing the two modalities is from many years ago (1980 and 1990s), and has less relevance given the improvement in quality of both imaging techniques since their introduction to clinical practice. These small historical studies comparing CT with MR imaging for pituitary adenomas showed CT to be reliable at detecting larger pituitary macroadenomas [10, 11] but inter-observer agreement is higher with MR [12]. Dedicated CT pituitary scans can provide sufficient evaluation for pituitary macroadenomas as well as providing some ability to detect larger microadenomas [13]. CT pituitary scans can also provide additional information that MR scans do not in terms of calcification or any bony destruction caused by the lesion [13]. They are often required for surgical planning [7].

Limitations of this audit include its small sample size, and that it was conducted in a single centre with dedicated Neuroradiologists. Taking this into account, the findings of our audit should be used for guidance only and do not replace clinical decision making. If a clinician believes an MR scan is required due to specific aspects of a patients’ case, then this should be requested without delay.

Another limitation is that those patients who had a normal CT pituitary scan did not have an MR pituitary scan done to confirm the normal finding and that no macroadenoma was present. However, it is arguable whether it is ethical both at an individual patient level, and more broadly at a population level, to perform an expensive test with a long waiting time when the pre-test probability of a clinically relevant finding on MR following a normal CT is likely to be extremely low.

## Conclusion

In summary, CT of the pituitary is a suitable option for initial pituitary imaging in the setting of the key clinical question being to exclude a pituitary macroadenoma. However, because of their better resolution and lack of ionising radiation, MR pituitary will remain the gold standard imaging technique for pituitary imaging, especially when detecting a small adenoma is of clinical value (for example in Cushing’s disease). The Auckland City Hospital Endocrinology Department now routinely requests CT pituitary scans when wanting to exclude a pituitary macroadenoma. We believe that this simple change in practice might be suitable for other hospitals, especially with the ongoing coronavirus pandemic and worsening pressure on MR waiting lists.

## Data Availability

The datasets used and analysed during the current study are available from the corresponding author on reasonable request.

## Abbreviations

CT: Computerised Tomography
KPI: Key Performance Indicator
mIU/L: Milli-international units per litre
nmol/L: Nanomoles per litre
MR: Magnetic Resonance
mSv: Millisievert (unit of radiation)
PACS: Picture Archiving Communication System
